# Applying fluorescent dye assays to discriminate *Escherichia coli* chlorhexidine resistance phenotypes from porin and *mlaA* deletions and efflux pumps

**DOI:** 10.1038/s41598-022-15775-6

**Published:** 2022-07-15

**Authors:** Branden S. J. Gregorchuk, Shelby L. Reimer, Carmine J. Slipski, Kieran A. Milner, Shannon L. Hiebert, Daniel R. Beniac, Timothy F. Booth, George G. Zhanel, Denice C. Bay

**Affiliations:** 1 Department of Medical Microbiology and Infectious Diseases, University of Manitoba, Winnipeg, MB Canada; 2 National Microbiology Laboratory, Public Health Agency of Canada, Winnipeg, MB Canada; 3 Department of Medical Microbiology and Infectious Diseases, University of Manitoba, Rm 514C Basic Medical Sciences Bldg, 745 Bannatyne Avenue, Winnipeg, MB R3E 0J9 Canada

**Keywords:** Biophysical chemistry, Microbiology techniques, Bacteria, Bacteriology, Infectious-disease diagnostics

## Abstract

Bacterial resistance to the antiseptic chlorhexidine (CHX), is a growing problem, recently shown to be caused by deleterious mutations to the phospholipid transport system component (*mlaA*) as well as efflux pump overexpression. Comparisons of CHX resistance mechanisms, such as porin deletions (*ompCF*), and over-expressed efflux pumps (*acrB, qacE, aceI*), are lacking and may be distinguishable using antiseptic rapid fluorescent dye testing assays. Using *E. coli* K-12 CHX adapted isolates (CHXR1), gene deletion mutants, and over-expressed transformants the phenotypes of these CHX resistance genes were compared using antimicrobial susceptibility tests (AST), rapid fluorescent propidium iodide dye-based membrane integrity assays (RFDMIA), and scanning electron microscopy (SEM). AST findings showed CHXR1, Δ*acrB*, Δ*ompCF*, and transformants pCA24N-*aceI* and pCA24N-*mlaA* conferred greater (two to fourfold) MIC changes when compared to matched controls. Examination of these mutants/transformants using CHX RFDMIA showed that porin dual-deletions (Δ*ompCF*) and *mlaA* alterations (Δ*mlaA*; pCA24N-*mlaA,* CHXR1) were distinguishable from controls. Results for over-expressed (pMS119EH-*aceI*) and deleted (Δ*acrB*) efflux pump RFDMIA could not be distinguished with propidium iodide, only with ethidium bromide, suggesting propidium iodide is better suited for detecting porin and *mlaA* associated CHX resistance mechanisms. SEM of CHXR1 and unadapted *E. coli* cells exposed to increasing CHX concentrations revealed that CHX does not visibly damage cell envelope integrity at any tested concentration but did identify elongated CHXR1 cells. Δ*mlaA* confers similar levels of CHX resistance as efflux overexpression and porin deletions, however, only outer membrane-altering porin and *mlaA* deletions can be reliably distinguished using RFDMIA.

## Introduction

Chlorhexidine (CHX) salts, commonly formulated as chlorhexidine digluconate, are widely used cationic antiseptics for healthcare and dental applications. It is an essential medicine recognized by the World Health Organization^1^ and listed in the top 300 most prescribed medications in the United States as of 2020^2^. Its mechanism of action primarily involves cell membrane disruption, where divalent cations at the outer membrane surface are displaced, allowing CHX to enter the membrane bilayer and form gaps between adjacent lipid headgroups including lipopolysaccharides^3^. CHX actions rapidly increases lethal cell and ion content leakage and protein denaturation in a concentration-dependent manner^4,5^. Although there are no currently established guidelines to define CHX resistance breakpoints from the Clinical Laboratory Standards Institute (CLSI) or European Committee on Antimicrobial Susceptibility Testing (EUCAST), reports of bacterial CHX resistance are increasing among clinically relevant Gram-negative bacterial species^5–13^. Bacterial CHX resistance is often correlated with antiseptic over-usage/contamination and its common addition to many personal hygiene and cleaning products^14^. CHX is a growing concern due to its frequent co-association with species that have resistance to peptide antimicrobials such as colistin, and ceragenins in Enterobacteria^8,13,15^, suggesting CHX may promote antimicrobial cross-resistance^16^.

The most well-established CHX resistance mechanisms in Enterobacteria are associated with efflux pump activities. Efflux-mediated CHX resistance mechanisms can involve: (i) The over-expression of intrinsic efflux pumps such as the dominant multipartite Resistance-Nodulation-Cell Division (RND) family *E. coli/Salmonella* spp. AcrAB pump^17^. (ii) The acquisition of foreign single component efflux pumps from plasmids and mobile genetic elements, such as small multidrug resistance (SMR) family protein QacE^18^ and proteobacterial Antimicrobial compound efflux (PACE) protein AceI^19,20^. In addition to efflux, CHX resistance mechanisms are speculated to alter outer membrane drug permeability caused by the loss or down-regulation of general diffusion porins such as OmpC/F^5,21^, however, porins have never been directly examined for CHX resistance. A recent multi-omics study of in vitro CHX-adapted *E. coli* K-12 identified that *ompF* was significantly down-regulated in the transcriptomes and proteomes of CHX-adapted *E. coli* isolates (CHXR1)^22^. In the same multi-omics study, the loss of an outer membrane lipoprotein MlaA was also implicated^22^. MlaA forms a complex with OmpC/OmpF^23^ and is part of the Mla intermembrane phospholipid transport system that maintains lipopolysaccharide asymmetry in the outer membrane^24^. Deletion of *mlaA* was shown to decrease CHX resistance by fourfold in CHX-adapted *E. coli*^22^ and other in vitro CHX adaption studies in *E. coli*^25^. The mechanism of Δ*mlaA* resistance is hypothesized to reduce CHX binding to lipopolysaccharides enriched in the outer membrane by increasing phospholipid accumulation. *MlaA* is the outer membrane associated lipoprotein anchor component of the intermembrane spanning maintenance of lipopolysaccharide asymmetry (MlaFEDCAB) system. The Mla system is responsible for removing phospholipids from outer leaflet of the outer membrane to maintain LPS enrichment^26,27^. Δ*mlaA* likely increases phospholipid accumulation in the outer membrane which reduces CHX-mediated LPS binding and cell disruption. Presently, it is unclear how these different mechanisms of resistance compare or what their altered cell membrane consequences yield on overall cell integrity during CHX exposure.

Recently, we developed a high-throughput rapid fluorescent-dye detection assay to discriminate antiseptic resistance phenotypes associated with quaternary ammonium compound antiseptics^28^. This assay known as rapid fluorescent propidium iodide dye-based membrane integrity assay (RFDMIA) may be useful for detecting various CHX resistance mechanisms in Gram-negative species in addition to quaternary ammonium compound (QAC) antiseptic resistance^﻿28^. RFDMIA can identify changes in bacterial cell membrane permeability caused by increasing QAC antiseptic exposure and its resistance by monitoring the changes in fluorescent emission of an impermeant fluorescent dye, propidium iodide (PI) over time. This 30-min assay measures cell membrane damage caused by antiseptic indirectly by monitoring PI fluorescence. Hence, as antiseptic concentrations increase, more antiseptic damage to the membrane promotes greater PI dye cell entry and consequently, greater fluorescent dye emission when PI binds to previously inaccessible intracellular DNA/RNA^29^.

This study compares how efflux pumps, porins, and *mlaA* alterations influence CHX resistant phenotypes and cell integrity in the *Escherichia coli* K-12 derivative BW25113 strain. To accomplish this each CHX resistance mechanism was examined using a recently described antiseptic RFDMIA^28^. A better understanding of how these resistance mechanisms compare is needed to predict how mutants and acquired resistance genes may influence antiseptic permeability across the cell membrane. This study identifies which resistance mechanism(s) can be reliably detected by RFDMIA. Here, we compared a CHX-lab adapted *E. coli* isolate (CHXR1) to wildtype *E. coli* BW25113 (WT). We also included a collection of single and dual gene deletion mutants (Δ*mlaA*^30^, Δ*acrB*^30^, Δ*ompCF*) and plasmid transformants expressing cloned genes (pCA24N-*mlaA*^31^, pMS119EH-*qacE*, pMS119EH-*aceI,* pCA24N-*acrB*^31^) to compare CHX resistance phenotypes. *E. coli* was prioritized, as it remains one of the top three antimicrobial-resistant blood and urinary tract infections^32,33^. We specifically chose the antimicrobial-susceptible *E. coli* K-12 derivative strain BW25113 due to its well characterized genetics. We performed broth microdilution antimicrobial susceptibility testing (AST) to determine and compare CHX minimum inhibitory concentration (MIC) values of ﻿*E. coli* with efflux, porin, and lipid alterations. To explore how these alterations influence CHX cell permeability, we utilized our recently developed RFDMIA, that discriminates antiseptic resistant bacteria based on differences in fluorescent dye uptake and emission intensity from antiseptic susceptible controls^28^. Lastly, to visually assess how CHX disrupts cell membrane integrity, we compared cell morphology differences of CHX-adapted *E. coli* (CHXR1) to its unadapted WT counterpart at increasing CHX exposures using scanning electron microscopy*.* This study provides a comprehensive comparison of different CHX resistance mechanisms in *E. coli*. It highlights the advantages and limitations of using RFDMIA for discriminating different CHX resistance mechanisms and explores the cell morphology alterations caused by Δ*mlaA* increasing CHX exposure in *E. coli*.

## Results

### AST of *E. coli* mutants, plasmid transformants, and CHX adapted isolates reveal that porin, lipid transporter and efflux pump gene alterations have similarly increased MIC values

To verify CHX resistance phenotypes of the various *E. coli* strains prior to RFDMIA analysis, AST was performed with *E. coli *gene deletion mutants, plasmid transformants, and CHX-adapted isolates to determine their CHX MIC values (Table 1). As previously reported^22^, AST of CHX-adapted *E. coli* CHXR1 demonstrated a fourfold increase in CHX MIC values when compared to the unadapted *E. coli* BW25113 strain (Table 1). Prior genetic analyses of CHXR1 in a recent study revealed that this adapted isolate possesses numerous gene mutations, including deleterious mutations to *mlaA* and its upstream promoter^22^. As shown in Table 1, only plasmid complementation with a wildtype copy of *mlaA* reverted this isolate back to CHX susceptible phenotypes observed for the unadapted WT^22^. AST of *E. coli* Δ*mlaA* (JW2343-KC) demonstrated a twofold increase in its CHX MIC value, however, BW25113 transformants over-expressing *mlaA* (pCA24N-*mlaA*) conferred a fourfold reduction in CHX MIC values when compared to its respective controls (Table 1). The lower CHX MIC value of Δ*mlaA* as compared to CHXR1, is likely due to the presence of other gene mutations in the adapted CHXR1 isolate as noted in its recent multi-omic analysis study^22^. In CHXR1, *fimE*, *gadE*, and *cdaR* all demonstrated minor (twofold) CHX MIC changes when they were either individually complemented in CHXR1 or they were examined as single *E. coli* gene deletion mutants^22^.

**Table 1:** A summary of CHX AST *E. coli* K-12 isolates, mutants and transformants MIC and 30MBC values determined in this study.

*E. coli* strain/isolate tested	Plasmid transformed	CHX MIC (µg/mL)	CHX 30MBC (µg/mL)^a^
BW25113 (WT)	–	2	32
	pMS119EH^b^	2	32
	pCA24N^c^	2	32
	pMS119EH-*aceI*	**8**	64
	pMS119EH-*qacE*	2	32
	pCA24N^c^-*acrB*	4	64
	pCA24N^c^-*mlaA*	**0.5**	**8**
CHXR1^d^	–	**8**	64
JW2343-KC (Δ*mlaA*)^e^	–	4	64
JW0451-KC (Δ*acrB*)^e^	–	**0.5**	16
JW0912-KC (Δ*ompF*)^e^	–	2	32
JW2203-KC (Δ*ompC*)^e^	–	2	32
KJ740 (Δ*ompC,* Δ*ompF*)^f^	–	4	32

^a^30MBC; 30-min minimal biocide concentration.

^b^pMS119EH; Ampicillin-selective P-tac expression vector as described in^52^.

^c^pCA24N; Chloramphenicol-selective T5/*lac* expression vector lacking the green fluorescent protein fusion tag as described in^31^.

^d^CHX-adapted BW25113 isolate 1, resistant to 9.6 µg/mL CHX after 20 sub-cultures^22^.

^e^Single genome deletion strains obtained from BW25113 as part of the Keio collection^30^.

^f^Strain obtained from the Yale Coli Genetic Stock Centre (https://cgsc.biology.yale.edu/) as KJ740 (AW740 and generated for the study by^34^. It is derived from the parental *E. coli* K-12 strain AW607 which when AST has the same CHX MIC and 30MBC as WT BW25113.

Significant values are given in bold.

To determine the role of porins in CHX resistance, we performed AST of *E. coli* Δ*ompF* (JW0912-KC) and Δ*ompC* (JW2203-KC). Neither single porin gene deletion strain showed any differences in CHX MIC values when compared to the WT (Table 1). Hence, the loss of either porin individually does not enhance CHX resistance. As both OmpC and OmpF can compensate for the loss of one another and also form an outer membrane complex with MlaA^23^, the deletion of both genes may be necessary to observe a CHX-resistant phenotype. Thus, we repeated CHX AST with *E. coli* KJ740 strain, which possesses a deletion of both *ompC* and *ompF*^34^. KJ740 AST results showed a twofold increase in CHX MIC values as compared to the WT (Table 1). This indicates that the loss of both porins had a low effect on CHX MIC values, similar to the Δ*mlaA* strain (Table 1). Given both porins are known to associate with MlaA in the outer membrane as a heteromeric complex^23^, our CHX MIC results for the dual KJ740 (Δ*ompCF*) strain and JW2343-KC (Δ*mlaA*) strain reaffirm previous findings supporting an MlaA-OmpC/F dependence.

Lastly, we performed AST of efflux pump-mediated CHX resistance mechanisms in *E. coli*. We began by examining *E. coli* lacking its dominant RND *acrB* efflux pump component (JW0451-KC; Δ*acrB*) and BW25113 over-expressing *acrB* (pCA24N-*acrB*; Table 1). Our AST results for Δ*acrB* (JW0451-KC) showed a fourfold reduction in CHX MIC values as compared to WT. When *acrB* was over-expressed, these transformants demonstrated only a twofold increase in CHX MIC value from the WT (Table 1). These findings suggest a requirement to express both *acrA* and *acrB* genes together for higher CHX MIC values in *E. coli,* as this pump is a multicomponent efflux complex. Overall, our AST findings show that the loss of *acrB* enhances CHX susceptibility to similar MIC values we observed for *mlaA* over-expression and that AcrB confers a modest level of resistance similar to porin and *mlaA* alterations (Table 1).

As most recognized CHX-selective efflux pumps are acquired from plasmids/integrons in *E. coli* and other Enterobacteria^18,20^, we also performed AST of transformants expressing *qacE* and *aceI* (Table 1). Transformants expressing SMR pump *qacE* (pMS119EH-*qacE*) showed no difference in CHX MIC values from the WT (Table 1). This finding was identical to recently reported *qacE* CHX MIC findings^18^. In contrast, WT transformants expressing PACE member *aceI* via pMS119EH, conferred a fourfold increase in CHX MIC value as compared to WT (Table 1). This finding was in agreement with CHX MIC values reported for *aceI* in a previous study^20^ and reconfirmed its role in CHX resistance.

Therefore, only CHXR1 (an *mlaA* mutant) and pMS119EH-*aceI* transformants appear to confer similar modest (fourfold MIC) levels of CHX resistance in *E. coli* when all mechanisms were compared. Conversely, the loss of *acrB* and the over-expression of *mlaA,* both similarly reduced CHX MIC by fourfold (Table 1). Altogether, AST indicates that the phenotypes of different CHX resistance mechanisms we tested, efflux pump over-expression (*aceI*), dual porin deletions (Δ*ompCF*), and altered expression of the lipid transport system component *mlaA*, each resulted in similar levels (two to fourfold MIC value) of CHX resistance in *E. coli*.

### RFDMIA can discriminate differences in PI dye emission for CHXR1 isolates, Δ*mlaA*, and *mla*A-over-expression strains when compared to controls

**Figure 1:**
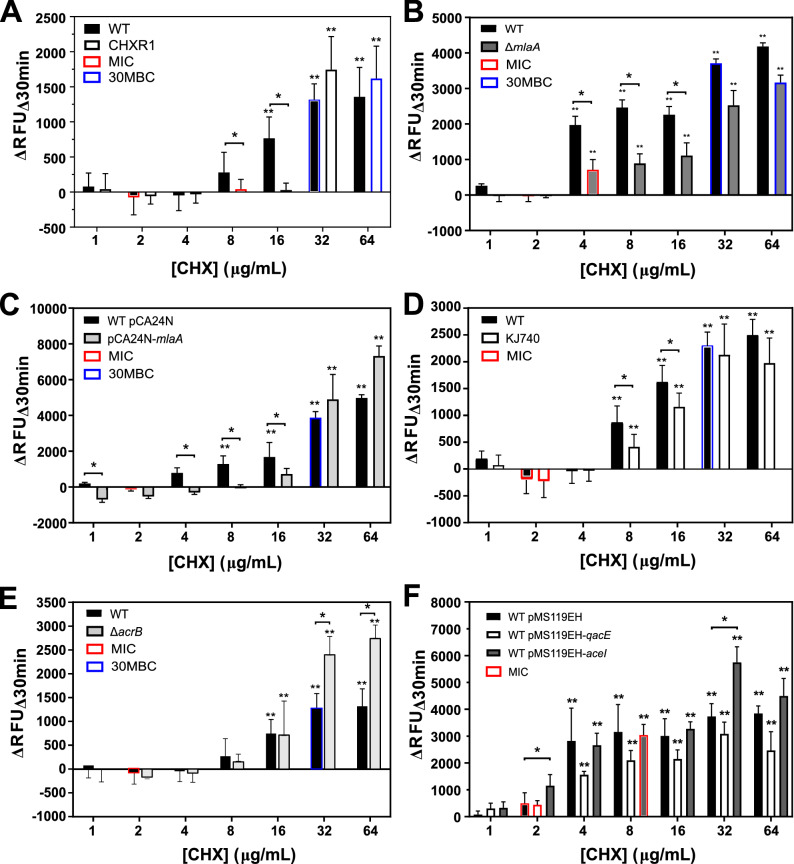
RFDMIA of *E. coli* K-12 BW25113 (WT), CHX-adapted isolate (CHXR1), single gene deletion mutants and plasmid transformants exposed to increasing CHX concentrations. In each panel, the mean ΔRFU_Δ30 min_ value at tested CHX concentration ranges of 1–64 µg/mL are shown for: (**A**) RFDMIA of WT and CHXR1 isolates, (**B**) Δ*mlaA* (JW2343-KC) and WT, (**C**) WT pMS119EH-mlaA and pMS119EH transformants, (**D**) KJ740 (Δ*ompC*, Δ*ompF*) and WT, (**E**) Δ*acrB* (JW0451-KC) and WT, (**F**) WT pMS119EH-*qacE*, pMS119EH-*aceI* and pMS119EH transformants. Statistical analysis of each RFDMIA plot involved Mann–Whitney U tests and was used to identify the lowest CHX concentration with a significant increase in ΔRFU_Δ30min_ value (**) for the same sample with *P*-values (*P* < 0.05). This test was also used to determine *P* < 0.05 for significantly different ΔRFU_Δ30min_ value comparisons between the control and the measured sample at the same CHX concentration and is denoted as horizontal lines with an asterisks (*). Bar plots represent a minimum of three bacterial bioreplicated cell suspensions measured in three technically replicated samples (n = 9).

RFDMIA results comparing each of the various *E. coli* gene mutants and overexpressed gene transformants to their appropriate controls is shown in Fig. 1. RFDMIA of CHXR1 isolates showed significantly lower ΔRFU_Δ30min_ values at 8–16 µg/mL CHX when compared to the WT (Fig. 1A). This shows that CHXR1 membranes were significantly less permeant to PI dye as compared to WT when exposed to the same CHX concentrations. These low ΔRFU_Δ30min_ values for CHXR1 occurred at CHX concentrations that were above the WT MIC but below the 30-min minimal biocide concentration (30MBC) value of WT and CHXR1 respectively (Fig. 1A). This finding indicates that RFDMIA is useful for discriminating outer membrane lipid perturbing mechanisms, since the CHX adapted isolate has deleterious *mlaA* mutations^22^, and *mlaA* can significantly alter inner to outer membrane phospholipid flux^26,27^.

Next, we examined if RFDMIA discriminated differences in PI emission in the Δ*mlaA* (JW2342-KC) gene deletion strain since CHXR1 has multiple gene alterations including *mlaA* that may confound its results. RFDMIA of JW2342-KC demonstrated low and statistically significant reductions in ΔRFU_Δ30min_ values at 4–16 µg/mL CHX when compared to WT (Fig. 1B). These RFDMIA findings agree with the differences in ΔRFU_Δ30min_ CHX concentration range values for CHXR1 when as compared to JW2342-KC results (Fig. 1A,B). RFDMIA of pCA24N-*mlaA* transformants showed a different trend in ΔRFU_Δ30min_ values at increasing CHX concentrations (Fig. 1C). Similar to previous RFDMIA findings for QAC-adapted Enterobacterial isolates^28^, pCA24N-*mlaA* transformants demonstrated statistically significant, negative ΔRFU_Δ30min_ values at 1–2 µg/mL CHX concentrations when compared to pCA24N transformants (Fig. 1C). This indicates that RFU_Δ30min_ values from cells over-expressing *mlaA* without added CHX result in greater PI dye emission when compared to WT. It also means there is greater PI dye permeability and penetration into *mlaA* expressing cells. As pCA24N-*mlaA* transformants were more susceptible to CHX based on their fourfold lower MIC values from the control (Table 1), RFDMIA findings for pCA24N-*mlaA* suggest that the outer membrane is destabilized by enhanced MlaA accumulation and more susceptible to CHX. Above 2 µg/mL CHX (or WT MIC), RFDMIA ΔRFU_Δ30min_ values of the WT were significantly reduced or negative at 4–16 µg/mL CHX when compared to its control (pCA24N transformant) (Fig. 1B,C). Hence, the results of pCA24N-*mlaA* transformants show that both Δ*mlaA* and MlaA over-accumulation phenotypes in *E. coli* were distinguishable from their respective controls when monitoring changes in PI dye emission at increasing CHX concentrations by RFDMIA.

### RFDMIA can discriminate differences in PI dye emission when comparing porin mutants to the wildtype but not for efflux pump mechanisms

RFDMIA analysis of the KJ740 (Δ*ompC*, Δ*ompF*) strain showed significant reductions in ΔRFU_Δ30min_ values at 8–16 µg/mL CHX values when compared to the WT (Fig. 1D). Reduced ΔRFU_Δ30min_ values for KJ740 occurred above CHX MIC and 30MBC values suggesting that PI penetration was most prominently detected in this CHX concentration range by RFDMIA. This finding is also in good agreement with previous studies of porin gene deletions that have shown reduced impermeant fluorescent dye penetration for dye compounds such as Hoechst H33342^35^. It is important to note here that PI is significantly larger than Hoechst H33348 (452.56 Da), ethidium bromide (394.29 Da), or 1-N-phenylnaphthylamine (219.29 Da). A PI molecule is an analogue of ethidium bromide (668.39 Da) with a +2 net charge and this molecule exceeds the known 600 Da size limit of OmpC/F porins^36^. When considering these properties, PI may be a better impermeant molecule for measuring membrane related damage caused by antiseptic actions as the size of PI dye exceeds known porin molecular sieving size limits, which can act as a confounding variable with smaller impermeant dyes used for porin permeation studies. Hence, *mlaA* alterations (mutant and overexpression) and Δ*ompC/F* porins mutants confer similar RFDMIA values at higher CHX values 4–16 µg/mL, suggesting the alteration of both porins that form an outer-membrane protein complex impacts CHX resistance.

RFDMIA results for efflux-mediated CHX resistance mechanisms was less pronounced and limited to particular CHX concentrations (Fig. 1E,F). Regarding efflux-mediated CHX resistance determinants, RFDMIA of Δ*acrB* (JW0451-KC) also demonstrated no significant differences in ΔRFU_Δ30min_ values at any CHX concentration tested, with the exception of 32 and 64 µg/mL CHX; at these concentrations a significant increase in JW0451-KC ΔRFU_Δ30min_ values was noted when compared to WT (Fig. 1E). Interestingly, JW0451-KC resulted in a fourfold reduction of CHX MIC when compared to WT (Table 1), yet this CHX susceptible mutant did not appear to affect PI dye penetration by RFDMIA. Since PI dye is larger (668 Da) than known OmpF/C porin sieving limits (600 Da)^37,38^, and porins were not altered in the Δ*acrB* efflux mutant, additional PI dye permeation and emission caused by its general diffusion was not anticipated in this mutant until values exceeding the WT CHX 30MBC (32 µg/mL) were reached, as we observed (Fig. 1E).

No significant differences in ΔRFU_Δ30min_ values were noted for any over-expressed efflux pumps pMS119EH-*qacE* or pMS119EH-*aceI* at any CHX value tested by RFDMIA (Fig. 1F). The only exception was pMS119EH-*aceI* transformants at 2 µg/mL and 32 µg/mL CHX, where each CHX concentration showed a significant increase in ΔRFU_Δ30min_ values as compared to the control pMS119EH transformant (Fig. 1F). It is notable that 2 µg/mL CHX is the WT CHX MIC value and 32 µg/ml CHX is well above the 30MBC for both the control and pMS119EH-*aceI* transformants (Table 1; Fig. 1F). Considering that significant differences in ΔRFU_Δ30min_ values were not detected at other CHX concentrations tested, it was unclear why PI emission did not increase at any intermediate CHX values (between 2 and 32 µg/mL CHX) by pMS119EH-*aceI* transformants. When we tested if PI dye itself may act as an AceI substrate, we could not accurately determine a PI MIC value, since WT cells exposed to PI dye concentrations were viable well above 0.5 mg/mL PI and exceeded accurate AST measurements due to saturating levels of dye. It is also important to note that both *aceI* and *qacE* efflux gene transformants were expressed from plasmids using a leaky P*tac* promoter. This pMS119EH plasmid was previously shown to confer non-toxic efflux pump expression at levels that conferred detectable CHX MIC value differences from the control vector^18,39^. Isopropyl β-d-1-thiogalactopyranoside (IPTG) induction of both efflux gene transformants above 0.05 mM concentrations have been previously shown to be toxic, reducing or arresting cell growth when added to media^18,20^; hence, we avoided IPTG induction in our AST and RFDMIA experiments as CHX resistant phenotypes were apparent (Table 1).

**Figure 2:**
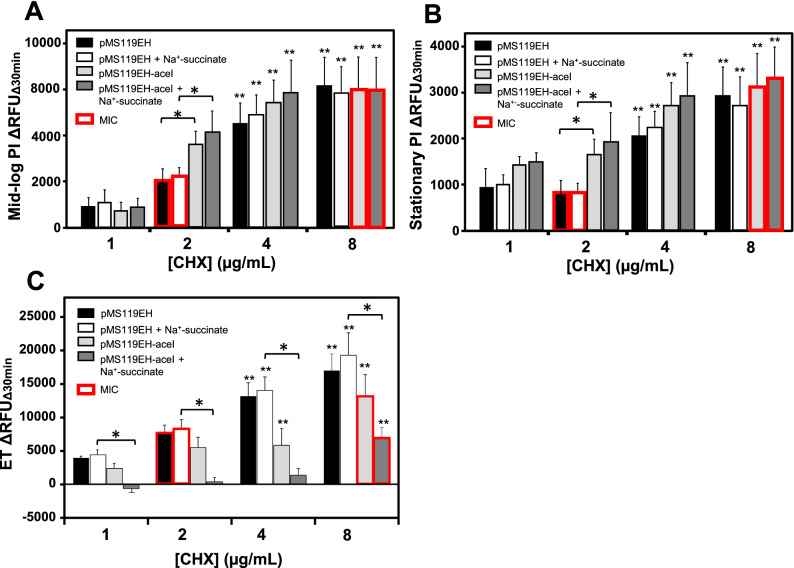
RFDMIA of *E. coli* K-12 BW25113 pMS119EH and pMS11EH-*aceI* transformants measured at mid-log or stationary phase with and without Na^+^-succinate and RFDMIA replacing PI with ET. (**A**) RFDMIA mean PI DRFU_D30min_ values of WT pMS119EH and pMS11EH-*aceI* transformants harvested at mid-log phase (OD_600nm_ = 0.5) at increasing CHX concentrations with and without 5 mM Na^+^-succinate addition. (**B**) RFDMIA mean PI ﻿ΔRFU﻿_Δ__30min_ values of WT pMS119EH and pMS11EH-aceI transformants harvested at stationary phase (OD_600nm_ = 1.1–1.5) at increasing CHX concentrations (1–8 µg/mL CHX) with and without 5 mM Na^+^-succinate. (**C**) RFDMIA mean ET ΔRFU_Δ30min_ values of WT pMS119EH and pMS11EH-*aceI* transformants harvested at stationary phase (OD_600nm_ = 1.1–1.5) at increasing CHX concentrations (1–8 µg/mL CHX) with and without 5 mM Na^+^-succinate. Mann–Whitney U tests were used to identify the lowest CHX concentration with a significant increase in ﻿ΔRFU_Δ30min_ value (**) for the same sample with *P*-values (*P* < 0.05). This statistical test was also used to determine *P* < 0.05 for significantly different ﻿ΔRFU_Δ30min_ value comparisons between the control and the measured sample at the same CHX concentration denoted as horizontal lines with an asterisks (*). In all panels, bar plots represent at minimum of three bacterial bioreplicate cell suspensions measured from three technical replicate samples (n = 9).

Lastly, since RFDMIA was unable to reliably discern CHX efflux mechanisms in *E. coli*, it was important to determine if this was due to the RFDMIA measurement conditions used. All previous RFDMIA analyses used stationary phase cells (Fig. 1). Stationary phase cells were selected for their speed and accuracy in past RFDMIA after a comparison of stationary and mid-log phase cells^28^. AcrAB and AceI are both secondary active proton motive force driven pumps that are most active when cells are growing with a respirable carbon source^19,40^. Hence, a lack of discernable PI dye ﻿ΔRFU_Δ30min_ differences between WT and efflux mutants/transformants may highlight the need for cell efflux to be measured at mid-log phase and/or in the presence of a respirable carbon (succinate or glucose), as described in previous fluorescent dye assays^35,41,42^. We repeated RFDMIA assays of *E. coli* pMS119EH-*aceI *and pMS119EH (control) transformants grown to mid-log and stationary phase with and without Na^+^-succinate supplementation (Fig. 2A,B). No significant differences in ΔRFU_Δ30min_ values for pMS119EH-*aceI* or the pMS119EH controls were noted under any of these suspension conditions in the presence of any CHX concentrations tested (Fig. 2A,B). However, when PI dye was exchanged for ethidium bromide (ET) in repeated RFDMIA of *E. coli* pMS119EH-*aceI* and pMS119EH transformants with and without Na^+^-succinate addition, significant reductions in pMS119EH-*aceI* ET ΔRFU_Δ30min_ values were only noted when Na^+^-succinate was added to assays (Fig. 2C). At CHX values of 1–8 µg/mL we observed that only pMS119EH-*aceI* ET ΔRFU_Δ30min_ values gradually increased as CHX increased (Fig. 2C). The gradual increase in ET ΔRFU_Δ30min_ values by pMS119EH-*aceI* in the presence of Na^+^-succinate only is likely explained by efflux pump substrate competition between ET and CHX by the AceI pump as ET is a renowned efflux pump substrate^42^. This finding indicates that only Na^+^-succinate energized *E. coli* transformants expressing *aceI* reduced ET emission when compared to cells lacking *aceI* genes. It should be noted that PI dye (668.4 Da) has a larger molecular weight than ET (394.29 Da). Therefore, this analysis also shows that ET can permeate across *E. coli* membranes more readily than PI, likely due to their size and net charge differences that favor ET for general diffusion porin entry (max sieving limit of 600 Da). Overall, ET is a more suitable dye for efflux-mechanism detection over PI when using RFDMIA.

### SEM analysis of WT and CHXR1 *E. coli* exposed to increasing CHX concentrations show that high CHX concentrations do not rupture cells but does alter cell morphology.

**Figure 3:**
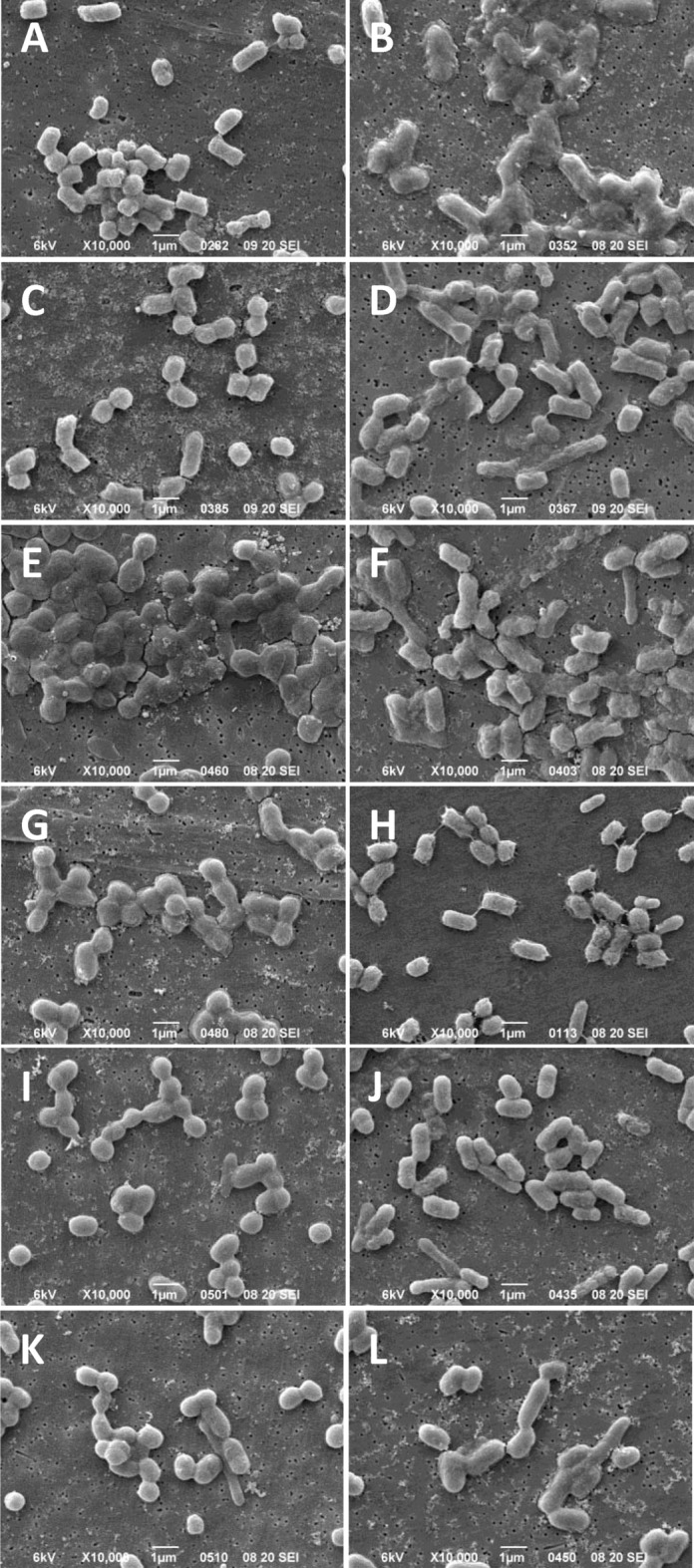
SEM images of *E. coli* K-12 BW25113 WT and CHXR1 replicates after 30-min exposure to increasing concentration of CHX. (**A**,**C**,**E**,**G**,**I**,**K**) shows WT cells and (**B**,**D**,**F**,**H**,**J**,**L**) show CHXR1 cells after 30-min exposure to CHX at 0 µg/mL (**A**,**B**), 1 µg/mL (**C**,**D**), 2 µg/mL (**E**,**F**), 10 µg/mL (**G**,**H**), and 32 µg/mL (**I**,**J**), 64 µg/mL (**K**,**L**). All images are representative of 5 SEM images collected at ×10,000 magnifications and the white scale bar at the bottom of each panel image indicates 1 µm lengths. Images from the same bioreplicated culture preparation are shown.

Membrane disruption caused by CHX has been speculated to destabilize and disrupt overall cell membrane integrity, therefore, we performed scanning electron microscopy (SEM) of CHXR1 and WT cells to visualize CHX-induced cell disruption. Here, we used *E. coli* cells prepared and exposed to the same CHX concentrations used in the 30-min RFDMIA (Fig. 3; Table 2). SEM cell images at 1000× magnification were used to determine if CHX exposure resulted in quantifiable reductions in total cell counts. Mean cell counts from 4 separate cell images, each exposed to 1 to 64 µg/mL CHX showed no statistically significant increases or decreases in total cell numbers when comparing WT or CHXR1 (Table 2). Additionally, all CHX concentration exposures to WT and CHXR1 showed a typical bacilliform morphology (Fig. 3). This indicates that CHX exposure, even at concentrations at or above the 30MBC values (WT 30MBC; 32 µg/mL, CHXR1 30MBC; 64 µg/mL) did not reduce the number of detectable bacilliform cells. This finding is in stark contrast to SEM images of QAC antiseptic exposed WT cells at or above their 30MBC which showed significant reductions in the total number of visible cells^28^. Hence, exposure of WT and CHXR1 cells to the antiseptic CHX at 30MBC concentrations does not visibly perturb cell envelope integrity.

**Table 2:** Mean cell counts with standard deviation (SD) for WT and CHX-adapted *E. coli* (CHXR1) cells visualized and averaged from 4 different 1000× magnification SEM images per sample at increasing CHX concentrations after 30-min of drug exposure.

CHX concentration (µm/mL)	WT (BW25113) ± SD	CHXR1 ± SD	*P*-value^a^
0	627 ± 41	547 ± 62	0.286
1	560 ± 54	584 ± 23	0.635
2	363 ± 86	475 ± 29	0.293
10	497 ± 45	455 ± 83	0.306
32	451 ± 75	495 ± 38	0.551
64	499 ± 95	513 ± 125	0.915

^a^*P*-values were determined using a two-tailed Students t-test comparing WT to CHXR1 for each CHX concentration.

To determine how CHX-induced membrane disruption influenced the overall WT and CHXR1 cell morphologies (length, width, and cell appearance) at increasing CHX concentrations, SEM cell images at 5000× magnification were examined (Fig. 3). Length and width measurements of cells imaged by SEM across all CHX concentrations tested, showed that CHXR1 cells were longer on average (0.316 µm ± 0.137 µm) and wider (0.040 µm ± 0.105 µm) than unadapted WT (length 1.260 µm ± 0.0.85, width 0.791 µm ± 0.040) (Table 3). Next, as a qualitative analysis we examined WT cells without added CHX and these cells maintained a classic turgid bacilliform morphology (Fig. 3A). At sub-inhibitory (1 µg/mL) CHX concentrations, WT cells appeared to be increasingly flattened (Fig. 3C) and at CHX MIC values (2 µg/mL) (Fig. 3E), where 65.4% of WT cells displayed pitting/wrinkling and/or cell fusion at 10,000× magnifications (Fig. S1A,B). Images of CHXR1 cells at 0 µg/mL CHX had a deflated bacilliform cell appearance similar to WT cells imaged at 2 µg/mL CHX (WT MIC) values (Fig. 3B). As CHX concentrations increased from 1–2 µg/mL, CHXR1 cells appeared to become more inflated, and the presence of elongated cells were noted (Fig. 3D,F). As seen with WT at or above its CHX MIC value, CHXR1 at 10 µg/mL CHX showed cells that were deflated in appearance with distinct pitting and cell wrinkling that was more pronounced than observed for the WT (Fig. 3H; Fig. S1C,D). At higher CHX concentrations approaching 30MBC values, WT cells were more fused in appearance as compared to WT cells with no added CHX or at sub-inhibitory CHX concentrations (Fig. 3G,I,K). The same trend was noted for CHXR1 at concentrations above its MIC (Fig. 3H,J,L). Lastly, at the 30MBC value of CHXR1 (64 µg/mL) both *E. coli* strains had rounder cell shapes, although CHXR1 remained significantly longer and narrower than WT cell (Fig. 3K,L; Table 3). These results agree with previous SEM analyses of *E. coli* and other bacterial species exposed to CHX. In the study by Cheung et al*.*^43^, cell pitting, cell “dents”, and cell-to-cell fusions were described for *E. coli* and *B. subtillis* cells exposed to CHX, similar to what we observed in our SEM analyses (Fig. S1A,B). Another SEM study by Shalamanov^44^, which examined *Pseudomonas aeruginosa*, *Enterobacter cloacae*, and *Serratia marcescens* cells exposed to hibitane gluconate 20%, an antiseptic with CHX as the main active ingredient, showed heterogeneous populations of cells for each species that exhibited increased cell clustering/aggregation at inhibitory antiseptic concentrations similar to our findings. Hence, SEM cell images support our CHXR1 and WT RFDMIA findings, showing CHX exposure alters cell membrane integrity in the presence of CHX. SEM also confirmed that all concentrations of CHX tested do not lyse cells, but CHX may gradually perturb WT cell integrity as concentrations reach or exceeded CHX MIC values.

**Table 3:** A summary of the mean and standard deviation (SD) values of measured cell lengths and widths by SEM for WT and CHX-adapted *E. coli* (CHXR1) after 30-min of exposure to CHX.

[CHX] (µg/mL)	Isolate	Length (L)	SD	Length difference^b^ to WT	Width (W)	SD	Width difference to WT	L/W ratio	SD
0	WT	1.363	0.293	**0.294**	0.742	0.083	**0.129**	1.862	0.454
	CHXR1	1.657	0.337		0.871	0.124		1.941	0.469
1	WT	1.189	0.230	**0.450**	0.765	0.054	**− 0.048**	1.561	0.314
	CHXR1	1.639	0.356		0.717	0.070		2.309	0.557
2	WT	1.169	0.215	**0.517**	0.789	0.072	**− 0.046**	1.492	0.301
	CHXR1	1.686	0.373		0.835	0.134		2.067	0.559
10^a^	WT	1.299	0.246	**0.241**	0.829	0.072	− 0.005	1.582	0.345
	CHXR1	1.54	0.258		0.824	0.081		1.888	0.378
32	WT	1.196	0.186	**0.206**	0.847	0.096	**− 0.199**	1.429	0.269
	CHXR1	1.402	0.274		0.648	0.074		2.194	0.507
64	WT	1.342	0.241	**0.186**	0.773	0.051	**− 0.062**	1.562	0.341
	CHXR1	1.388	0.274		0.712	0.087		1.982	0.474

Values are based on images of individual cells visualized from five SEM images collected at 5000× magnification (n = 100 cells) for two bioreplicates.

All bolded values are significant with *P* < 0.01 by Student’s t-test.

^a^10 µg/mL was selected as this is the arithmetic MIC value.

^b^Difference from WT was determined by subtracting mean length or width of WT from the mean length or width values of CHXR1.

## Discussion

**Figure 4:**
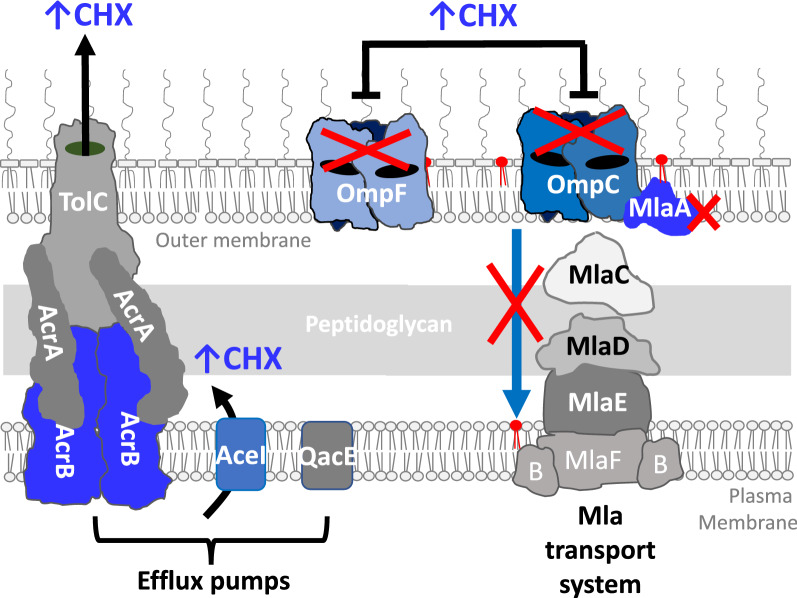
A diagrammatic summary of CHX resistance mechanisms compared in this study from the perspective of the *E. coli* cell envelope. Efflux pump systems, porins and MlaA as part of the intermembrane complex is shown. Complexes highlighted in blue show genes deleted (red X) or overexpressed in this study and their consequences for CHX susceptibility.

The AST findings of CHXR1, specific gene deletion mutants, and gene overexpression transformants in this study offers additional insight into intrinsic and acquired CHX resistance mechanisms. A diagrammatic summary of these CHX resistance mechanisms is provided in Fig. 4. The findings from our analysis show that the loss of both porins (*ompC, ompF*), the alteration efflux pump expression (*aceI,* Δ*acrB*), and the loss of outer membrane lipoprotein *mlaA* each independently confers similar CHX resistance phenotypes. Many of these findings are novel particularly with respect to OmpC/F porins and AcrB’s role in CHX resistance. These findings will help improve future resistance surveillance studies by characterizing genes that influence CHX susceptibility in *E. coli* and potentially other Enterobacterales species.

The application of the antiseptic screening assay RFDMIA to discriminate the various mutants and genes shown to alter CHX MIC values from WT controls also revealed important insights into the limitations and advantages of using PI dye to indirectly measure antiseptic-induced cell damage and infer CHX susceptibility. The combination of AST and RFDMIA findings for CHXR1, JW2343-KC (Δ*mlaA*) and pCA24N-*mlaA* offers greater insights into how *mlaA* contributes to CHX tolerance and membrane permeation from the recent study^22^. For the Δ*mlaA* strain, RFDMIA PI dye permeability was much lower than the WT but occurred at CHX values between WT MIC and 30MBC values (Fig. 1A,B, Table 1). This makes RFDMIA useful for discriminating membrane alterations caused by lipid-altering resistance mechanisms at relevant CHX concentrations. Over-expression of pCA24N-*mlaA* resulted in cells that were more permeant to PI dye without added CHX, and more susceptible to CHX (MIC at 0.5 µg/mL; Table 1). This finding suggests that cells accumulating more MlaA may enhance CHX susceptibility potentially due to imbalance of phospholipids and lipopolysaccharides in the outer membrane caused by a malfunctioning Mla retrograde lipid transport system.

Additionally, over-accumulation of MlaA in the outer membrane may increase MlaA-OmpC/F complex formations^23^, which could disorganize overall outer membrane integrity^23,24,26,27,45–47^. Our RFDMIA findings showed that WT strains over-expressing MlaA promoted greater CHX membrane disruption at much lower CHX concentrations than the WT (Fig. 1)^23,24^. Since CHX’s mechanism of action binds to and perturbs liposaccharide and phospholipid head groups to form gaps^4^, the Mla system may be an ideal transport system for lipid-bound CHX to gain entry into the cell. Since the Mla system is responsible for trafficking phospholipids from the outer to inner plasma membrane^26,27^, MlaA is an ideal entry point for lipid-binding antiseptics like CHX to exploit and for CHX resistance mechanisms to emerge from (Fig. 4). Our findings also suggest that the deletion of both OmpC/F porins is necessary to alter CHX MIC values from WT (Table 1; Fig. 1). Since the loss or downregulation of *ompC* and *ompF* often occurs in antibiotic resistant species^38^, porin downregulation may be another transcriptionally regulated factor that contributes to overall CHX-resistance. Porins influence cross-resistance to other antimicrobials due to reduced outer membrane drug permeability (as reviewed by^36^).

This study revealed that CHX-resistant efflux pump mechanisms (*aceI, qacE,* and *acrB*) in *E. coli* could not be discriminated by RFDMIA using PI dye. AceI-mediated resistance could only be discerned from controls when PI was replaced with ET in mid-log cultured cells, and only when a respirable carbon source (Na^+^-succinate) was added to the cell resuspension buffer (Fig. 2A–C). Therefore, PI dye is mainly suitable for rapidly detecting and screening antiseptic resistant mechanisms that specifically alter outer membrane function and integrity, *i.e*. alterations of *mlaA* and *ompC/F* porins. Proton driven efflux pumps such as AceI and AcrAB that act within the inner plasma membrane when cells are growing^19,40^, rely on antiseptic substrate entry and permeation under energetic conditions. Since PI dye is too large too diffuse through porins, none of the altered growth conditions we tested (stationary vs. mid-log with or without Na^+^-succinate) showed that PI is a suitable dye to discriminate efflux mechanisms (Fig. 2). ET on the other hand would not be suitable for screening *mlaA* or porin deletion mutants as ET is much smaller than PI may still be diffusible through other porins (OmpW, YddB and PhoE)^38,48^.

Our study highlights the utility of two different fluorescent dyes PI and ET to rapidly screen for different antiseptic resistance mechanisms. PI is a structural analog of ET, where it has an additional quaternary aminopropyl group at its the central pyridine ring, giving PI a net + 2 cationic charge at neutral pH and ET a net + 1 charge. Size and charge differences of both dyes demonstrated that PI is ideally suited for screening membrane-specific antiseptic disrupting resistant mechanisms such as porin and Mla intermembrane transport system mutants. As previously shown in many efflux pump assays^42^, ET fluorescent dye is more membrane permeant than PI due to its smaller size and reduced positive charge making it more suitable for measuring efflux-mediated mechanisms. Hence, both fluorescent dyes are useful for screening different antiseptic resistant mechanisms that alter drug efflux (ET) or disruption of the membrane (PI). The need for cells to be physiologically active to energize secondary active proton motive force driven efflux pumps is another factor to consider that may limit RFDMIA for screening efflux-mediated mechanisms, as highlighted in our findings using PI or ET with and without Na^+^-succinate added (Fig. 2C).

SEM analyses of WT and CHX-adapted *E. coli* influenced cell membrane integrity and morphology in the presence of CHX in two important ways (Fig. 3). First, SEM images collected from *E. coli* CHXR1 and WT cells confirmed that even at the highest CHX exposure, neither cell preparation was visibly ruptured. This indicates that CHX-mediated *E. coli* membrane perturbation differs from other cationic antiseptics such as QACs^28^. Secondly, CHXR1 known to possess with deleterious *mla*A mutations^22^, were differentiated by significant cell elongation as CHX exposure increases when compared to the WT, which may be used to distinguish the *ΔmlaA* phenotype in future studies.

Lastly, it is important to note that CHXR1 and individual genetic alterations in *E. coli* studied herein that enhanced CHX resistance, only resulted in low-level, fourfold increased MIC changes (Table 1). These low-level CHX MICs are typical of most known CHX resistant genotypes that we and others have previously reported^49,50^. The mutants/overexpression strains we examined only conferred resistance to CHX up to 4–8 µg/mL and are not expected to have significant impacts on CHX resistance when this antiseptic is used at its high recommended working concentrations (100–10,000 µg/mL; 0.01% w/v–10% w/v CHX) in topical oral mouth washes/rinses and skin ointments/washes^1^. However, low-level CHX-resistant species may become more consequential when introduced to CHX-exposed environments (mucosa, disinfected surfaces) with residual levels of inhibitory CHX. Under these conditions, low-level CHX resistant species can be selected for and out compete CHX susceptible species, as suggested from past clinical studies^5,49–51^ or even enhance antibiotic cross-resistance^16,51^.

In conclusion, due to CHX usage becoming more widespread in a wider variety of products especially since the COVID-19 pandemic, understanding the mechanisms of intrinsic and acquired CHX resistance is paramount. Our AST analyses of *E. coli* resistance genes show that the loss of *mlaA,* the loss of both porins (Δ*ompF* Δ*ompC*), and expression of efflux pump *aceI* conferred CHX resistance (two or fourfold from WT), and efflux pump Δ*acrB* mutants conferring greater CHX susceptibility from the WT (Table 1). RFDMIA findings suggest that MlaA, part of the Mla intermembrane lipid transport system both outer membrane porins OmpC/F are important CHX cell access points (Figs. 1, 4). Our findings also show that PI dye in RFDMIA cannot be used to discriminate efflux-mediated CHX resistance mechanisms (*aceI*, *qacE* and *acrB*). This is likely due to the charge and size of PI dye exceeding general diffusion porin sieving limits even when cells are energized with a respirable carbon source to drive pump activity. As there is no consensus on antiseptic breakpoints and no rapid testing methods for discriminating CHX and antiseptic tolerance, RFDMIA offers an alternative approach to rapidly differentiate antiseptic resistance mechanisms in Enterobacteria and other bacterial species with PI and ET dyes. With further optimization and additional testing of Gram-negative and Gram-positive species, this rapid fluorescent dye assay not only has utility for future antiseptic mechanistic studies but also potential for targeted diagnostic surveillance.

## Materials and methods

### Chemicals, bacterial strains and culturing conditions.

Chlorhexidine digluconate (CHX; C9394) was purchased from MilliporeSigma (USA) and propidium iodide (P3566) was purchased from ThermoFisher Scientific (ON, Canada) all remaining chemicals were purchased from Avantor/VWR (Canada). For this study, an *E. coli* K-12 BW25113 isolate adapted to CHX from^22^ was compared to wildtype *E. coli* control (WT). The adapted isolate was generated using a repeated sub-culturing method that involved gradual increases in CHX concentrations over 20 subcultures resulting in a final adapted strain with fourfold higher MIC values when compared to the original unadapted WT strain^22^. *E. coli* K-12 single gene deletion strains JW2343-KC (Δ*mlaA*) and JW0451-KC (Δ*acrB*) were obtained from the Keio collection^30^ and strain KJ740 (Δ*ompCF*^34^) was obtained from the Yale Coli genetic Stock Centre. The genes *qacE* (NC_001735) and *aceI* (EII93401) were cloned into the multiple cloning site of the ampicillin selectable P*tac* expression vector pMS119EH described by Fürste et al.^52^ at restriction enzyme cut sites, XbaI and PstI (*qacE*) and XbaI to HindIII (*aceI*). Both genes were constructed and cloned using gene synthesis and subcloning services from BioBasic Inc (ON, Canada). Cryopreserved ASKA collection library strains MGNA-AG1, JW0451-AM, and JW2342-AM were used to isolate pCA24N, pCA24N-*acrB*, and pCA24N-*mlaA* plasmids respectively and were extracted from overnight cultures with chloramphenicol selection using BioBasic plasmid DNA extraction kits (BioBasic Canada). All plasmid DNA was subsequently transformed into BW25113 competent cells with appropriate selection to maintain plasmids using a standard RbCl_2_ chemical transformation protocol^53^. Sanger sequencing was used to verify all gene sequences. SDS-Tricine polyacrylamide (12–16% acrylamide) gel electrophoresis was used to confirm membrane protein over-expression by transformants using the methods described in Slipski et al.^23,24,54^. CHXR1 isolates were verified to be *E. coli* K-12 based on 16S rDNA polymerase chain reaction PCR amplification with Sanger sequencing and whole genome sequencing analyses^22^. All isolates were grown in Luria Bertani (LB) broth at 37 °C in a shaking incubator at 170 RPM, where many isolates, mutants and transformants were also grown in LB with appropriate antimicrobial selection: 2 µg/mL CHX for CHXR1 isolates, 100 µg/mL ampicillin for pMS119EH transformants, and 30 µg/mL chloramphenicol for pCA24N transformant selection).

### Antimicrobial susceptibility testing (AST)

The broth microdilution AST method described by Balouiri et al*.*^55^ was selected to measure antimicrobial susceptibility of our *E. coli* isolates. Briefly, cryopreserved bacterial stocks were grown overnight, where the OD_600nm_ of these cultures were standardized to a final OD_600nm_ of 1.0 unit with a DU530 spectrometer (Beckman and Coulter, USA). This standardized culture was diluted to 1:10,000 into each well of a 96-well microplate (167008; Thermo Scientific, USA) containing LB broth and log_2_ dilutions of antimicrobial. Inoculated plates were incubated in shaking incubator at 37 °C and 170 RPM where the OD_600nm_ was measured after 18 h growth with a Multiscan™ spectrum microplate reader (Fisher Scientific, USA). MIC values were defined as the lowest concentration with no discernable bacterial growth when compared to uninoculated control (LB + antimicrobial) wells. Mean MIC values were determined from 3 biological and 3 technical replicates.

### RFDMIA measurements of *E. coli* strains/isolates

The RFDMIA method described by Gregorchuk et al.^28^ was performed. Briefly, bacteria were grown overnight (18 h) in LB from a cryopreserved stock and standardized to an optical density at 600 nm (OD_600nm_) of 2.0 units with a DU530 spectrophotometer (Beckman and Coulter, USA). Cells were pelleted by centrifugation at 14,000 RPM for 1 min, washed in 0.2 µm filtered phosphate buffered saline (PBS), and then resuspended to final concentration of OD_600nm_ of 0.4 units for the assay. For mid-log phase cell preparations, 18 h overnight cultures were substituted with 1/100 diluted subcultures in LB that were grown at 37 °C with shaking to mid-log OD_600nm_ values of 0.5–0.6 units. 96-well black walled fluorescent microplates (12-566-72; Thermo Scientific, USA) were prepared with 2.0 µg/mL PI in each well and increasing concentrations CHX. Modified RFDMIA where Na^+^-succinate was added to assays involved the addition of filter sterilized Na^+^-succinate to a final concentration of 5 mM. For assays requiring ET, PI was excluded and replaced with ET at a final concentration of 4.0 µg/mL ET (10 µM ET); ET values were determined from commonly used efflux assays^42^. Standardized buffered cell suspensions were then diluted in each well to a final OD_600nm_ 0.2 units per well. A total of 3 biological and 3  technical replicates were measured in the assay. On each assay plate, a set of “heat-treated” cell suspensions were prepared by boiling at cells for 30 min at 121 °C to generate dead cell controls to determine maximum dye emission (EM) values for the assay. Plates were immediately measured in a fluorescent plate reader (Polarstar Optima, BMG Labtech, Germany) with EM 620 nm values being measured in 5 min intervals over 30 min. The change in relative fluorescent units (RFU) was calculated as described by Gregorchuk et al.^28^ to determine the change in RFU over 30 min (ΔRFU_Δ30min_) which is detailed below. PI dye emission from samples containing no CHX antiseptic were subtracted from samples at increasing antiseptic concentrations and plotted as the change in relative fluorescent units (ΔRFU_Δ30min_; Fig. 1). After 30 min, the spot plate viability of each *E. coli* cell suspension was performed using a 48-pin replicator (04-450-10, Boekel Scientific, USA) to stamp 1–2 µl of sample onto LB agar plates. Three LB agar plates were examined per biological replicate, allowing us to determine the mean 30-min minimum biocidal concentration (30MBC). 30MBC was defined as the lowest antimicrobial concentration where no visible bacterial growth was observed on agar plates. 30MBC measurements were performed to determine CHX exposures that killed cells in 30-min RFDMIA to more accurately determine assay cell viability at various CHX concentrations tested. The change in PI RFUs at excitation of 490 nm and 620 nm emission after 30-min (ΔRFU_Δ30min_) was determined using the calculations as described in Gregorchuk et al.^28^. The change in ET RFUs was determined at an excitation of 530 nm and 600 nm emission was monitored over 30 min on a BioTek Synergy Neo2 Hybrid multimode microplate reader fitted with dual photomultiplier tubes (BioTek, CA, USA). ET ΔRFU_Δ30min_ values were calculated as described in Gregorchuk et al.^28^ for PI. When comparing any ΔRFU_Δ30min_ values between antiseptic-susceptible and resistant isolates, higher ΔRFU_Δ30min_ values at lower antimicrobial concentrations indicate a strain that is antiseptic susceptible; lower ΔRFU_Δ30min_ values at higher antimicrobial concentrations indicate a strain/species that is antiseptic resistant. Each ΔRFU_Δ30min_ value at a given drug concentration can be used to discriminate antiseptic susceptible from resistant bacteria^28^.

### Scanning electron microscopy (SEM)

To visualize the membrane disruptive capabilities of CHX on *E. coli* cells and confirm RFDMIA results, we utilized a scanning electron microscope (JCM-5700, JEOL USA, USA). Our bacterial samples were prepared for SEM using the “Gold sputtering” method described by Golding et al.^56^ with the modification that we diluted our bacteria 1:1000. In an effort to quantify WT and CHXR1 cell counts, cell length and width distributions, as well as overall cell turgidity after CHX exposure, we visualized 20 cells in different areas of 5 separately collected images on the filter at 1000×, 5000× and 10,000× magnifications. Cell counts were determined from 1000× magnification images, where cells were counted in grids using ImageJ V1.52a^57^ and the mean cell counts and standard deviations are reported in Table 2. For cell length and width measurements using 5000× images, we measured twenty bacterial cell lengths and widths per image (n = 100) with ImageJ V1.52a^57^, which were subsequently statistically analyzed (Table 3). To determine bacterial cell turgidity and appearance variations, SEM images were blinded and analyzed by three different study authors and were scored for the proportion of “inflated”, “deflated”, “indeterminate” bacteria. To better display the membrane disruptive capacities of CHX, higher magnification images (10,000×; Fig. S1) were selected as representative examples of WT and CHXR1 cell morphology from the 5000× magnification image analyses shown in Fig. 2.

### Statistical analyses

All statistical analyses were performed using GraphPad Prism V6 (GraphPad Software, USA) or Excel365 (Microsoft, USA). For SEM analysis, we performed two two-tailed Student’s t-tests with 4-sets of images collected from two biological replicates. Due to lower replicate sampling sizes, Mann–Whitney U statistical analysis tests were used to analyze all RFDMIA ΔRFU_Δ30min_ 620 nm emission data. Mann–Whitney U tests were used to compare the same strain at a given concentration of CHX to the lowest CHX concentration and considered changes with *P*-values of < 0.05 to be significant (shown as ** on RFDMIA figures). The second statistical analysis with Mann–Whitney U compared the ΔRFU_Δ30min_ values between isolates at the same CHX concentration (shown as * on RFDMIA figures), with *P*-values of < 0.05 considered to be significant. All RFDMIA values shown in Figs. 1-2 represent the mean ΔRFU_Δ30min_ of dye at given emission values and the standard deviations were determined from the mean of three technical replicates per biological replicate (total n = 3) of an isolate unless otherwise indicated.

## Supplementary Information

Supplementary Figure S1.

## Data Availability

The raw fluorescent emission spectral datasets and electron microscopy imaging generated and/or analysed during the current study is available from the corresponding author on reasonable request.
