# Supplementary material for: Applying fluorescent dye assays to discriminate Escherichia coli chlorhexidine resistance phenotypes from porin and mlaA deletions and efflux pumps
Source: Sci Rep. 2022 Jul 15;12:12149. doi: 10.1038/s41598-022-15775-6 (PMC9287405; doi:10.1038/s41598-022-15775-6)
Supplement: Supplementary file 1 — Supplementary Figure S1. [file 41598_2022_15775_MOESM1_ESM.pdf]

Supplementary files for the manuscript entitled:

Applying fluorescent dye assays to discriminate *Escherichia coli* chlorhexidine resistance phenotypes from porin and *mlaA* deletions and efflux pumps

A research article by:

Branden S.J. Gregorchuk[1], Shelby L. Reimer[1], Carmine J. Slipski[1], Kieran A. Milner[1], Shannon L. Hiebert[2], Daniel R. Beniac[2], Timothy F. Booth[2], George G. Zhanel[1], Denice C. Bay[1§].

1. Department of Medical Microbiology and Infectious Diseases, University of Manitoba, Winnipeg, Manitoba, Canada
2. National Microbiology Laboratory, Public Health Agency of Canada, Winnipeg, Manitoba, Canada

[§]Corresponding author:

Denice C. Bay
Assistant Professor
Rm 514C Basic Medical Sciences Bldg.
Department of Medical Microbiology and Infectious Diseases
University of Manitoba
745 Bannatyne Avenue
Winnipeg, MB, Canada R3E 0J9
Tel: (204) 977-5679
Fax: (204) 789-3926
Email: Denice.Bay@umanitoba.ca

Keywords: chlorhexidine, efflux pump, antiseptics, disinfectant, scanning electron microscopy, fluorescent spectrometry, porins, mlaA, aceI, acrB, propidium iodide, ethidium bromide

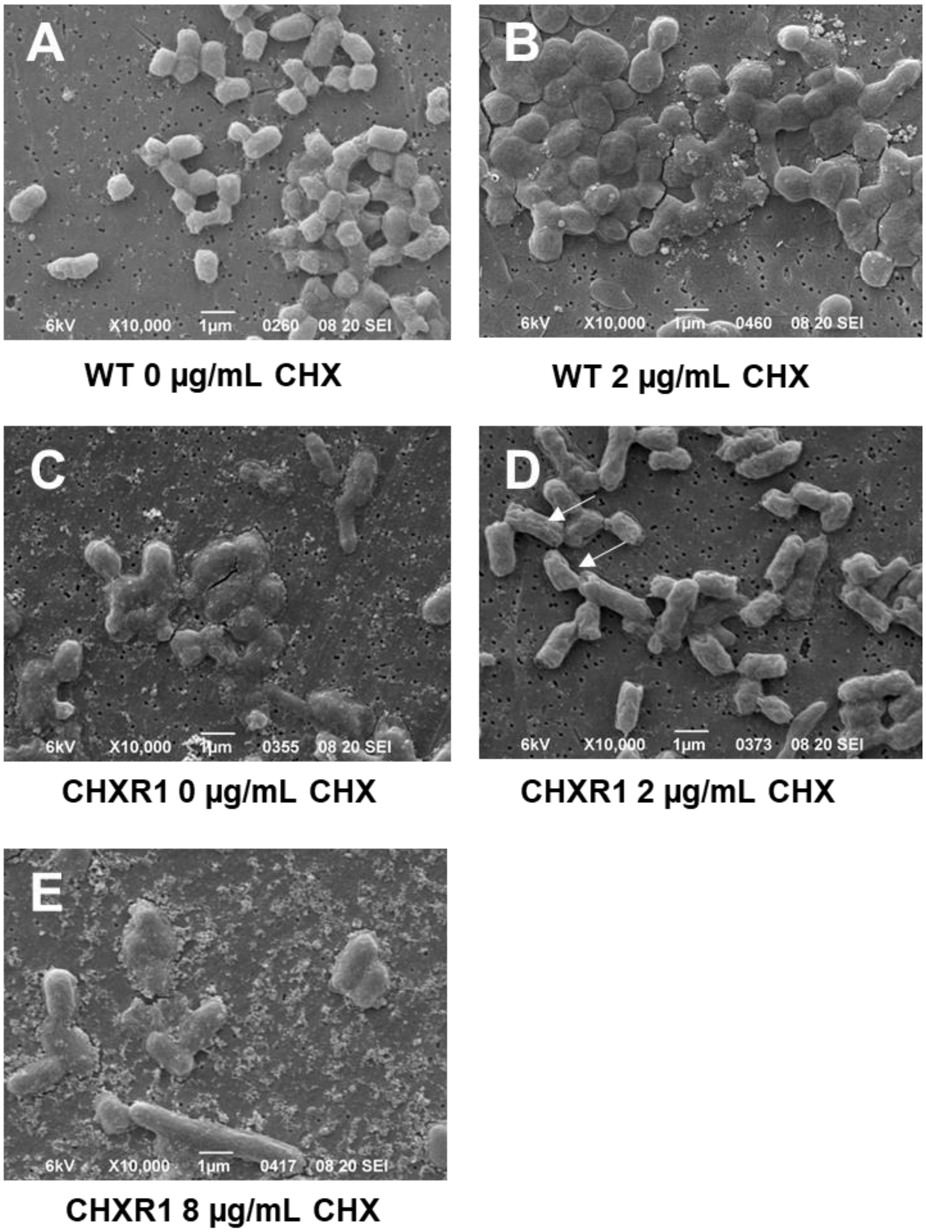

Figure S1. Representative 10,000X magnification SEM images of *E. coli* K-12 BW25113 WT and CHXR1 replicates after 30-minute exposure to their respective MIC CHX concentrations. Panel **A)** shows WT cells without CHX addition and panels **B**) shows the appearance of cells with 2 µg/mL CHX (MIC value) added. Panel **C)** shows CHXR1 without CHX (0 µg/mL) and panels **D-E** show CHXR1 cells at 2 µg/mL CHX (sub-MIC value; **D**) and 8 µg/mL CHX (arithmetic MIC value; **E**) respectively. Arrows on panel D show wrinkled pitting of CHXR1 cells. All images are a representative SEM image collected from 4 images at 10,000x magnification. The white scale bar at the bottom of each panel image indicates 1 µm lengths. Images from the same bioreplicated culture preparation are shown.
